# Impact of a Heat Shock Protein Impurity on the Immunogenicity of Biotherapeutic Monoclonal Antibodies

**DOI:** 10.1007/s11095-019-2586-7

**Published:** 2019-02-15

**Authors:** Shraddha S. Rane, Rebecca J. Dearman, Ian Kimber, Shahid Uddin, Stephen Bishop, Maryam Shah, Adrian Podmore, Alain Pluen, Jeremy P. Derrick

**Affiliations:** 1 School of Biological Sciences, Faculty of Biology Medicine and Health Manchester Academic Health Science Centre, The University of Manchester, Michael Smith Building Oxford Road, Manchester, M13 9PT UK; 2 Medimmune Ltd, Granta Park, Cambridge, CB21 6GH UK; 3 Medimmune, 1 Medimmune way, Gaithersburg, Maryland 20878 USA

**Keywords:** adaptive immunity, aggregation, host cell impurity, immunogenicity, monoclonal antibody

## Abstract

**Purpose:**

Anti-drug antibodies can impair the efficacy of therapeutic proteins and, in some circumstances, induce adverse health effects. Immunogenicity can be promoted by aggregation; here we examined the ability of recombinant mouse heat shock protein 70 (rmHSP70) - a common host cell impurity - to modulate the immune responses to aggregates of two therapeutic mAbs in mice.

**Methods:**

Heat and shaking stress methods were used to generate aggregates in the sub-micron size range from two human mAbs, and immunogenicity assessed by intraperitoneal exposure in BALB/c mice.

**Results:**

rmHSP70 was shown to bind preferentially to aggregates of both mAbs, but not to the native, monomeric proteins. Aggregates supplemented with 0.1% rmHSP70 induced significantly enhanced IgG2a antibody responses compared with aggregates alone but the effect was not observed for monomeric mAbs. Dendritic cells pulsed with mAb aggregate showed enhanced IFNγ production on co-culture with T cells in the presence of rmHSP70.

**Conclusion:**

The results indicate a Th1-skewing of the immune response by aggregates and show that murine rmHSP70 selectively modulates the immune response to mAb aggregates, but not monomer. These data suggest that heat shock protein impurities can selectively accumulate by binding to mAb aggregates and thus influence immunogenic responses to therapeutic proteins.

**Electronic supplementary material:**

The online version of this article (10.1007/s11095-019-2586-7) contains supplementary material, which is available to authorized users.

## Introduction

Monoclonal antibodies (mAbs) are the fastest growing sector of the global pharmaceutical industry, particularly for the treatment of cancer and inflammatory diseases [1,2]. Unwanted immunogenicity and the formation of anti-drug antibodies (ADAs) is a significant challenge for the use of biotherapeutics, even for those that have been humanized [3], impacting safety and efficacy. A variety of factors can influence immunogenicity: these include product-related factors such as protein conformation or impurities, patient-related variables such as immune and genetic background, disease status and treatment-related factors such as route and duration of exposure [4, 5]. Aggregation is an important factor which has been implicated in immunogenicity and its likelihood is increased with the use of mAbs at high concentrations [6–8]. The link between aggregation and enhanced immunogenic responses is well established in mouse models (including transgenic animals). For example, aggregate percentage and the extent of denaturation of interferon beta-1a (IFNβ-1a) have been shown to influence the ability of aggregates to break tolerance in transgenic mice [9]. Only aggregates that retained native epitopes were able to stimulate a transient immune response and their removal prevented the breakdown of tolerance [9]. Aggregation is thought to be initiated by association of partially unfolded conformational states which can occur at any stage of bioprocessing and storage. They range in size and dimensions in the 0.1-10 μm range have been identified as being the most immunogenic [10]. Characteristics of aggregates which may contribute to immunogenic potential include the formation of neo-epitopes, multiple valency, post-translational modifications, concentration and size [11–13]. Despite the wealth of evidence demonstrating that aggregation results in enhanced immunogenicity, the underlying mechanisms are incompletely understood [14].

The removal of host cell protein (HCP) impurities is an important problem in the isolation of biologics for clinical use [15]. HCPs originate from the expression host, most commonly Chinese Hamster Ovary (CHO) cells for monoclonal antibodies. Measurement of total HCP content is commonly conducted by ELISA: null cell line isolates are used to immunize mice and obtain polyclonal antisera. This method is limited, however, in that it is dependent on the total average response to a crude mixture of HCPs which will, individually, have differential abilities to stimulate antibody production [15]. HCPs have been shown to influence immunogenicity as antigens or adjuvants [16,17], through HCP-induced protease activity [18] or direct biological activity [17], highlighting the need for HCP identification and individual quantification.

Heat shock proteins (HSPs) are a common HCP impurity [19]; they participate in housekeeping functions and are part of responses to environmental stresses [20,21]. Their chaperone activity in macromolecular complex assembly, protein transport and degradation acts to stabilize and correct the folding of nascent polypeptides *de novo*, dissociating aggregates, and re-folding stress-denatured proteins [22]. Stresses can exacerbate protein conformational problems, exceeding the capacity of chaperone systems to prevent aggregation. In addition, the combined use of HSPs has been explored as a strategy for enhancing vaccine potency [23]. Studies have shown, for example, that HSP-peptide complexes successfully elicited MHC class I restricted cytotoxic T lymphocyte responses, whereas HSP or peptide alone were not immunogenic, establishing the tumor-derived HSP gp96 as the first adjuvant of mammalian origin [24]. HSP-based cancer vaccines, such as artificially reconstituted HSP peptide antigen complexes, have been widely exploited [25]. In addition, the adjuvant property of mycobacterial Heat Shock Protein 70 fusion protein has been demonstrated using a variety of model antigens [26]. Our laboratory has recently shown that low levels of an *E. coli* HSP, DnaK, were able to enhance the immunogenicity of a recombinant 25 kDa human single chain variable fragment (scFv) following immunization of BALB/c strain mice [27]. HSPs therefore have the potential to function as adjuvants.

The principal aim of the current investigation was to establish whether this adjuvant-like effect could also be observed with aggregated human biotherapeutic mAbs and a cognate mammalian HSP, similar to that found in CHO cells. To this end, we used recombinant mouse HSP70 (rmHSP70), an ortholog of *E. coli* DnaK which is 98% identical to CHO HSP70 [27]. We show that rmHSP70 binds preferentially to aggregates and is able to exert an adjuvant-like effect on immune responses in a BALB/c mouse model. The implications for the contribution of HCPs to the immunogenicity of therapeutic protein aggregates are discussed.

## Materials and Methods

### Animals

Female BALB/c strain mice (8–12 weeks old) were used for these experiments (Envigo, Bicester, UK). Mice were housed on sterilized wood bedding with materials provided for environmental enrichment. Food (Beekay Rat and Mouse Diet No1 pellets; B&K Universal, Hull, UK) and water were available *ad libitum*. The ambient temperature was maintained at 21 ± 2°C and relative humidity was 55 ± 10% with a 12 h light/dark cycle. All procedures were carried out in accordance with the Animals (Scientific Procedures) Act 1986, and approved by Home Office licence.

### Monoclonal Antibodies

Two human monoclonal antibodies were used for the current study, hereafter referred to as mAb1 and mAb2. mAb1 has a theoretical molecular mass of 148 kDa and an experimentally measured p*I* of 7.6. mAb2 (a bispecific antibody) has a theoretical molecular mass of 204 kDa and an experimentally measured p*I* of 9.1. Both the mAbs were provided by MedImmune (Cambridge, UK).

### Aggregate Formation and Spiking with rmHSP70

Purified mAbs were diluted into 1 mg/mL in Dulbecco’s phosphate buffered saline (DPBS) without Ca^+2^ or Mg^+2^ (Sigma-Aldrich, St Louis, Missouri). In order to form aggregates of mAb1 by thermal stress, it was treated at 60°C for 25 min. To generate mAb1 aggregates using shaking stress, the solution at 1 mg/mL was shaken in a bench top shaker at 3000 rpm for 12 h at 22°C. mAb2 aggregates were formed by shaking stress in the same way, but at 1500 rpm for 4 h at 22°C. rmHSP70-aggregate complex samples were prepared by addition of rmHSP70 (Enzo Life Sciences, UK) to 0.1% by mass into the mAb aggregate within 5 min of mAb aggregation. The aggregates formed were stable and did not dissociate into monomers when the temperature was subsequently decreased by refrigeration, or after storage at −80°C.

### Dynamic Light Scattering (DLS)

Measurements of DLS were performed using a Malvern Zetasizer Nano ZS ZEN3600 (Malvern, Herrenberg, Germany), equipped with a 633 nm laser. Each sample (70 μL) was measured in a Suprasil® quartz cuvette (Hellma GmbH, Muellheim, Germany) with a path length of 3 mm and 200–2500 nm spectral range. Monomeric and stressed samples at 1 mg/mL were measured at 25°C to determine the volume-based average protein particle diameter in solution.

### Raster Image Correlation Spectroscopy (RICS)

SYPRO® Red (Molecular Probes, Oregon) was prepared as a 50x stock solution in pre-filtered histidine-sucrose buffer and diluted to a final working concentration of 2.5x for fluorescence studies immediately prior to use (all solutions were prepared on the day of use) [28]. SYPRO® Red was added 15 min prior to visualization with confocal microscopy. A Zeiss 510 Confocor 2 (Zeiss, Jena, Germany) confocal microscope equipped with a c-Apochromat 40×/1.2NA water-immersion objective was used for image acquisition. Imaging was carried out by exciting the dye with a Helium-Neon laser at 543 nm and the emitted fluorescence collected above 585 nm (LP585 filter set). A confocal image time series of 1024 × 1024 pixel resolution was captured over 100 frames with a corresponding pixel dwell time of 6.4 μs. In-house RICS software (ManICS) was applied to analysis of images acquired using confocal microscopy. A full description of the RICS algorithm has been described elsewhere [28,29]. The image time series were sub-divided into 32 × 32 pixel sub-regions and the diffusion coefficients (D) of each region of interest (ROI) was generated. The method is described in greater detail previously [28].

### Asymmetric Flow Field Flow Fractionation (AF^4^)

Asymmetric flow field-flow fractionation (AF^4^) is used to measure aggregate content and molecular mass distribution, as an orthogonal method to size exclusion chromatography or analytical ultracentrifugation. In the current study, aggregates of mAb1 (generated by thermal stress) and mAb2 (generated by shaking stress) in the presence and absence of rnHSP70 were prepared as described above. Separation was achieved by a liquid cross-flow which takes place in a narrow, ribbon-like channel of trapezoidal geometry, which is built up by a spacer, between a porous and a nonporous plate. The porous plate is covered by a membrane, which acts as accumulation wall and allows the eluent to pass the membrane, while the particles/macromolecules are retained [30]. Water was used as solvent for the method at 25°C, with 30 min analysis time and 0.294 s sampling time. The total area of the peaks and the molecular mass of the rmHSP70-aggregates were measured. Corresponding monomer mAbs (with and without rmHSP70) were used for comparison. The ASTRA chromatography software (Wyatt Technology) was used to analyse the data.

### Fluorescence Microscopy

rmHSP70 (low endotoxin) was purchased and was obtained commercially labelled with BODIPY FL by Life Technologies, USA. BODIPY FL labelled rmHSP70 (0.1%) was added to the thermal stressed mAb1 and shaking stressed mAb2 aggregates. 2.5x SYPRO® Red dye was added to the aggregated mAb immediately before analyzing the sample using a Zeiss 510 Confocor 2 microscope.

### SDS Page

Protein samples were diluted in SDS-PAGE sample buffer (Bio-Rad, Berkley, CA, USA) containing 1% (*v*/v) 2-mercaptoethanol and heated for 5 min at 90°C. Samples were resolved on a pre-cast NuPAGE 4–12% Bis-Tris Protein gels (Invitrogen™) and stained using InstantBlue™ Coomassie protein stain (Expedeon, Swavesey, UK).

### Western Blot

Protein samples were resolved on pre-cast 4–12% acrylamide gel at various concentrations (0.1, 0.01, 0.001, 0.005 and 0.05 μg/mL in PBS), transferred onto nitrocellulose membrane and detected using anti-HSP70 horseradish peroxidase (HRP) conjugated antibody (StressMarq Bioscience Inc., Victoria, British Columbia, Canada) diluted at 1:2000. Proteins on the blots were visualized using enhanced chemiluminescence reagents (Thermo Scientific).

Monomer and aggregate samples were prepared as described previously. rmHSP70 was added at 1:1000 ratio by mass to monomer and aggregated mAbs. For monomeric samples, where pellets were not observed, 30 μl PBS was added to the tube to dissolve any sedimented material. Protein samples were resolved on a pre-cast 4–12% acrylamide gel and transferred onto a nitrocellulose membrane. The presence of rmHSP70 was detected using anti-HSP70 horseradish peroxidase (HRP) conjugated antibody and blots were visualized using enhanced chemiluminescence reagents, as described above.

### Immunizations

Mice (*n* = 3 or 5 per group) were immunized by intraperitoneal (I.P.) injection on days 0, 7 and 14 and were exsanguinated on day 21. Animals were immunized with 250 μL of 1 mg/mL of mAb1 and 150 μL of 1 mg/mL of mAb2 (monomeric or aggregated) in PBS with or without rmHSP70 at a ratio of 1 in 1000 (0.1% by mass) relative to the immunizing mAb immediately after aggregate formation. Individual serum samples were prepared and stored at −80°C until analysis.

### ELISA for Analysis of Serum from mAb Immunized Mice

To analyse the serum from mAb immunized mice, plastic Maxisorb® plates (Nunc, Copenhagen, Denmark) were coated with 0.1 mg/mL monomer or 0.05 mg/mL aggregate mAbs in PBS overnight at 4°C. Protein-coated plates were blocked with 2% (*w*/*v*) bovine serum albumin (BSA)/PBS (Sigma Aldrich) at 37°C for 30 min. Doubling dilutions of serum samples were added (starting dilution 1:140 for IgG, 1:1120 for IgG1, 1:35 for IgG2a and 1:70 for IgM) prepared in 1% BSA/PBS and plates incubated for 3 h at 4°C. Negative control naïve mouse serum (NMS) or PBS sham control mouse serum samples were analyzed concurrently. Plates were incubated for 2 h at 4°C with HRP labelled sheep anti-mouse IgG diluted 1:4000, sheep anti-mouse IgG1 diluted 1:2000 (both Bio-Rad) or goat anti-mouse IgM diluted 1: 6000 (Invitrogen, Paisley, UK), diluted in 1% BSA/ PBS. For IgG2a ELISAs, MCA1588P rat anti mouse IgG2a HRP-heavy chain antibody (Bio-Rad) was used for mAb1 and STAR133P goat anti mouse IgG2a HRP antibody (Bio-Rad) was used for mAb2 (both at 1:1000 dilution). Plates were washed between incubations with 0.05% Tween 20 in PBS. For color development, plates were incubated with substrate (1.6 mg/mL *o*-phenylenediamine and 0.4 mg/mL urea hydrogen peroxide in 0.5 M citrate phosphate buffer (pH 5)), 100 μL/well, for 15 min in the dark and reactions were stopped with 50 μL/well of 0.5 M citric acid. Absorbance was read at 450 nm using an automated reader (ELx800; BioTek Instruments, Inc., Winooski, US), using the Gen 5 1.10 software. Data are displayed with respect to antibody titre (log_2_) calculated as the lowest serum dilution at which a 3x serum blank OD450nm reading was reached.

### [^3^H]Thymidine Splenocyte Proliferation Assay

A single cell suspension of splenocytes from immunized mice was prepared using mechanical disaggregation. Splenocytes were stimulated *in vitro* with respective protein samples for 7 days. 5 × 10^4^ cells/well splenocytes were co-cultured with 5 × 10^3^ bone marrow derived dendritic cells (BMDCs) pulsed with the protein immunogen or no protein (for control) in quadruplicate in round-bottom 96-well plates [31]. [^3^H]thymidine incorporation proliferation assay method is described elsewhere [14]. Cultures were incubated for approximately 60 h at 37°C, 5% CO_2_ and [^3^H]thymidine (^3^HTdR) (PerkinElmer, Waltham*,* MA*,* USA*)* was added at 37 kBq/well for the final 18 h, plates were harvested onto glass fibre filter mats with a multichannel semi-automated harvesting device (Titertek, Skatron AS, Lierbyen, Norway) and quantified with β scintillation counting. Results are presented as counts per min (cpm) as means of quadruplicates as described previously [31].

### Measurement of IFNγ Secretion Using ELISpot Assay

Splenocytes from immunized mice were cultured *ex vivo* using mAb1 or mAb2. BMDCs prepared as described previously were used as Antigen Presenting Cells (APCs) [31]. ELISpot assays were performed according to the manufacturer’s protocol (Mabtec, Nacka Strand, Sweden). Aliquots of 5 × 10^4^ cells/well were assayed in triplicate. The plates were developed after 48 h with BCIP (5-bromo-4-chloro-3-indoyl phosphate p-toluidine salt) and NBT (p-nitro blue tetrazolium chloride) color development solution (Bio-Rad) for 30–45 min and plates were rinsed with tap water. Spots were quantitated with an ELISpot reader (Cellular Technology Limited, Bonn, Germany). An animal was scored as positive when the response in the peptide-containing well was at least twice that of control wells, as described previously [32].

## Statistical Analyses

Statistical analyses were performed using Graphpad Prism 7. Analysis of variance (ANOVA) was used to determine statistical significance of differences between groups. Experiments were analyzed by non-parametric one way or two way ANOVA followed by Tukey’s post hoc test (*p < 0.05, **p < 0.01, ***p < 0.001).

## Results

### Characterization of Therapeutic mAbs in Native and Aggregated States

**Fig. 1:**
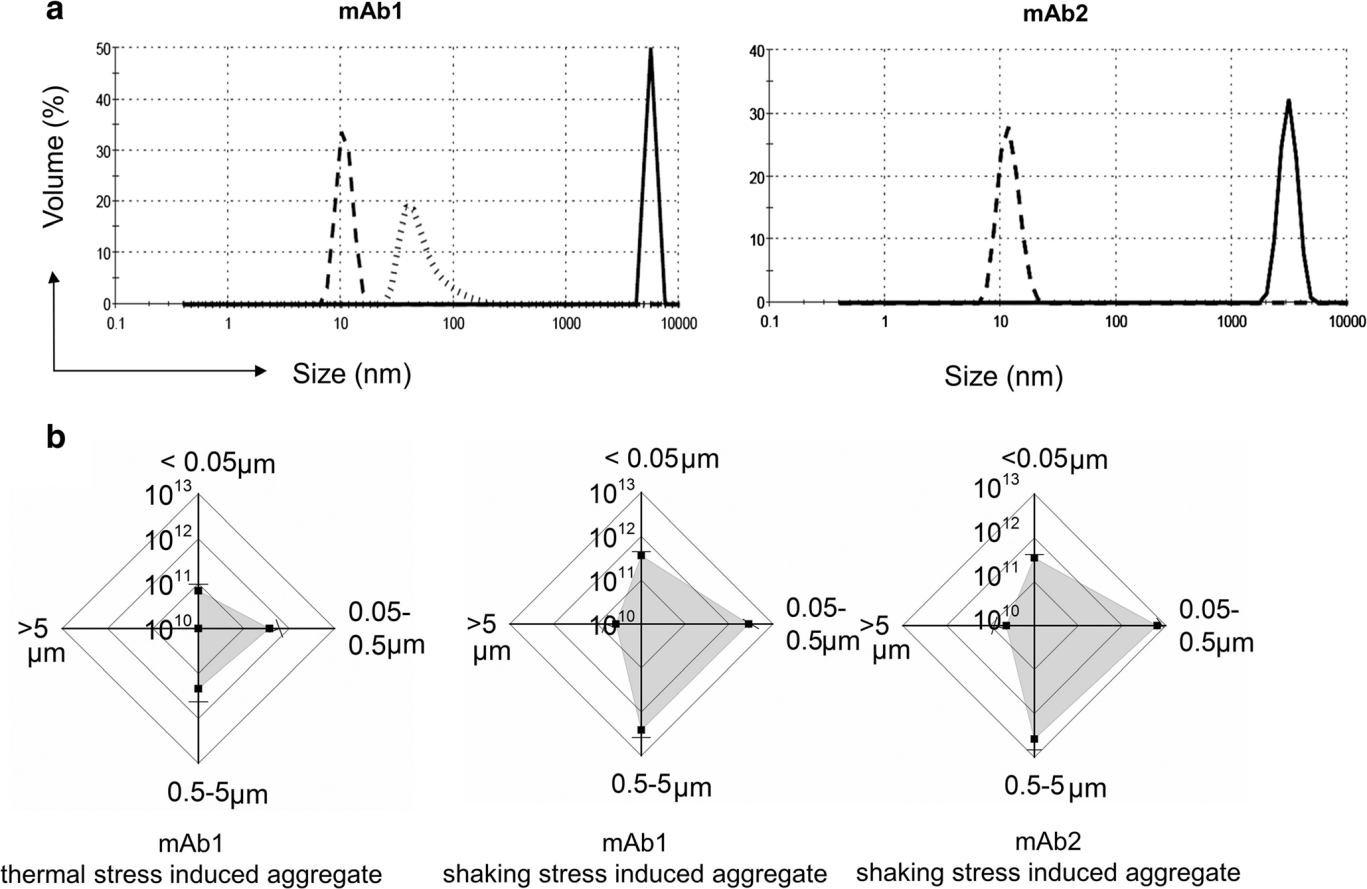
**Characterization of mAb1 and mAb2 aggregates by DLS and RICS.** (**a**) Aggregate particle size measured using DLS. Dashed line, monomer mAb; solid line, shaking stress induced aggregated mAb; dotted line, thermal stress induced aggregated mAb. (**b**) Analysis of aggregate species using RICS. Horizontal and vertical axes represent particle counts per mL. Data were separated into size ranges of <0.05, 0.05–0.5, 0.5–5 and > 5 μm. Values represent means ± SD (*n* = 3).

This investigation sought to investigate whether the adjuvant-like effect, observed previously using bacterial DnaK with aggregates of a scFv fragment [27], could also be recorded using intact monoclonal antibodies and a mammalian HCP. Consequently, two human IgG1 mAbs, mAb1 and mAb2, were used in the current study. Both mAbs were prepared at 1 mg/mL in PBS and aggregates generated by application of thermal or shaking stress. mAb2 showed no aggregation in response to thermal stress (at 45, 50 or 60°C) but aggregates of mAb1 were generated by both methods. The sizes of the generated mAb1 and mAb2 aggregates were analyzed by DLS (Fig. 1a). Both mAb monomers showed a narrow size distribution at ~10 nm, as anticipated (dashed line). Application of thermal stress to mAb1 generated an aggregate population within the sub-visible size range. Aggregate sizes were much larger (~1 μm) when formed by shaking stress for both mAb1 and mAb2 (Fig. 1a, solid line). For mAb1, different stress conditions (thermal stress, shaking stress, and stir stress) were found to give rise to different size populations of aggregate species, as analysed by DLS (Supplementary Fig. 1A). The mAb1 and mAb2 aggregates generated were irreversible and did not dissociate on dilution. In contrast to mAb1, mAb2 appeared more stable to different stresses applied and demonstrated different aggregation kinetics. Agitation stress was also applied to both mAbs using a tube rotator for up to 24 h, but failed to generate detectable aggregates (Supplementary Fig. 1). To characterize the aggregate populations more comprehensively, they were analyzed by Raster Image Correlation Spectroscopy (RICS). RICS provided a quantitative measurement of aggregate numbers (particles/fL) for four different size ranges, and for both mAbs (Fig. 1b). The results confirm that shaking stress caused aggregates to form within the 0.05–0.5 μm and 0.5–5 μm size ranges, a shift to larger size populations compared with the results of thermal stress induction on mAb1. These aggregate preparations were then used for an analysis of murine recombinant HSP70 (rmHSP70) binding and immunological responses.

### Binding of rmHSP70 to mAb1 and mAb2 Aggregates

**Fig. 2:**
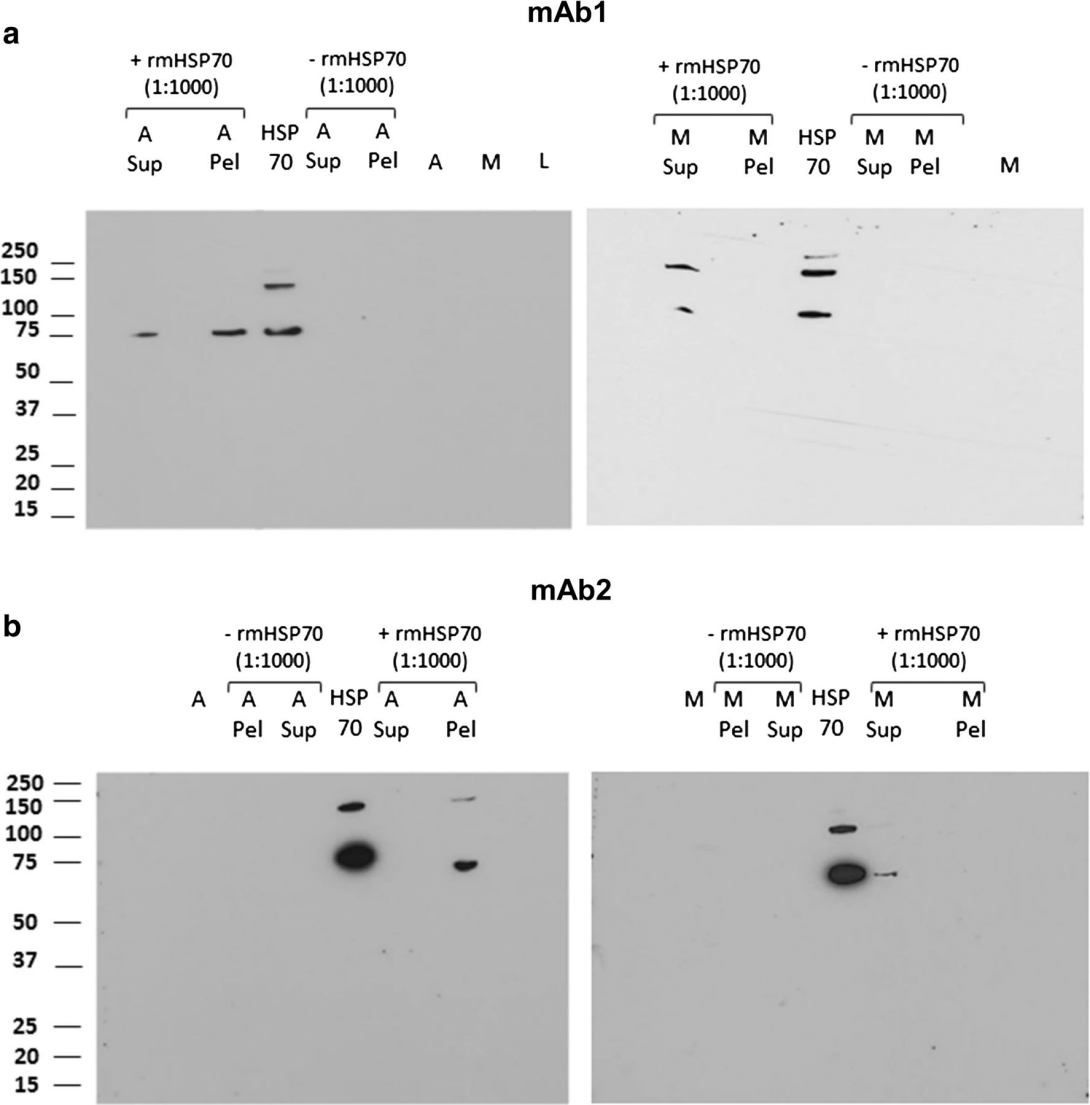
**Partition of rmHSP between aggregated and monomer fractions analyzed using western blot.** mAb1 and mAb2 were aggregated using thermal and shaking stresses, respectively. rmHSP70 was added at 0.1% by mass to monomer and aggregated mAbs immediately after aggregation. Samples were centrifuged at 12000 rpm for 30 min to sediment aggregated species; pellet and supernatants were then harvested. Blots were incubated with HRP conjugated anti rmHSP70 antibody and developed by chemiluminescence. Lanes are labelled as follows, M, monomer; A, aggregate; Sup, supernatant; Pel, pellet.

Our previous work has shown that the HSP DnaK was able to bind to scFv aggregates, a property which could contribute to its stimulation of immune response to aggregates [27]. We therefore conducted similar experiments to examine whether rmHSP70 was able to bind to aggregates of mAb1 and mAb2. rmHSP70 was added at 0.1% by mass with respect to mAb concentration to monomer or aggregated mAb1 and mAb2, insoluble aggregates were separated from monomer by centrifugation, and supernatant and pellet fractions were analyzed by western blotting using anti-rmHSP70 antibody (Fig. 2; SDS PAGE gel images are shown in Supplementary Fig. 2a and b). The partition of rmHSP70 was compared in the presence of monomeric mAb (right panels of Fig. 2a, b) or aggregated mAb (left panels of Fig. 2a, b). rmHSP70 alone migrated as a monomer (~75 kDa) or dimer (~150 kDa). For both mAb1 and mAb2, rmHSP70 selectively accumulated into the aggregated pellet fractions; this was not the case for monomeric mAb1 and mAb2, where rmHSP70 was exclusively found in the supernatant. We conclude that rmHSP70 selectively binds to mAb1 or mAb2 aggregates, rather than monomer.

**Fig. 3:**
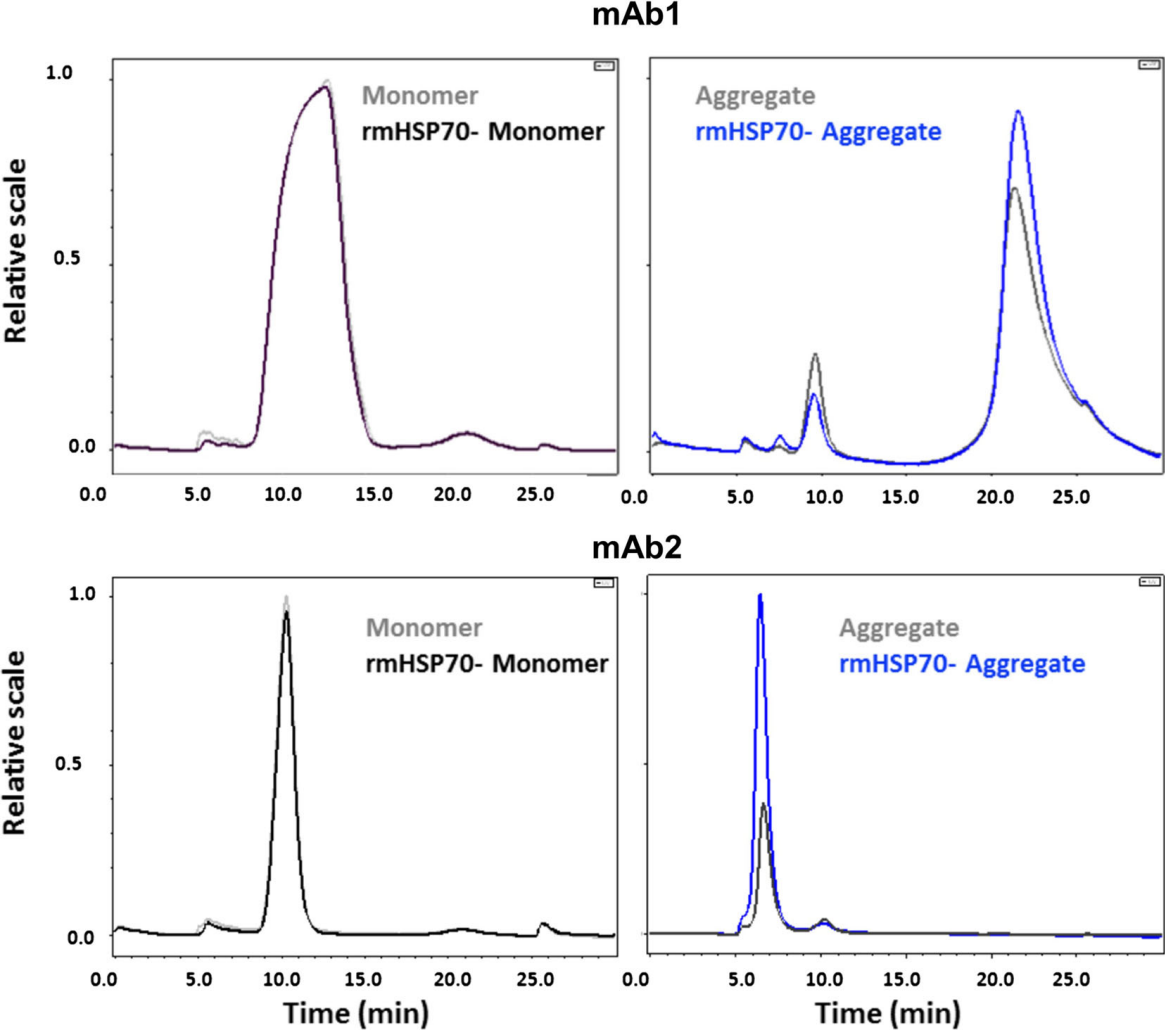
**Assessment of rmHSP70 binding to aggregated mAb using AF**^**4**^**.** rmHSP70 was added to the aggregated mAbs immediately after aggregation and binding was assessed by AF^4^. Chromatograms for mAb1 and mAb2 monomer (with and without rmHSP70) and aggregates (with and without rmHSP70) are overlaid as shown. The peak profiles were used to compile the data summarized in Table I (representative data from a single experiment).

Asymmetric Flow Field Flow Fractionation (AF^4^) was used as an additional method to examine rmHSP70 binding to the aggregated mAb preparations. Migration profiles of mAb1 and mAb2 monomers and aggregates were compared with and without rmHSP70 addition (Fig. 3). 50 μL of mAb1 and 10 μL of mAb2 samples were loaded to obtain optimum elution profiles; the heights, peak areas and elution times are shown in Table I. Addition of rmHSP70 did not significantly alter the elution times of monomers or aggregates but increased the peak area, by 22% for mAb1 aggregate and, remarkably, about 300% for mAb2 aggregate (Table I). No such effects were seen for addition of rmHSP70 to mAb monomers. We propose that these effects are caused by changes to aggregate properties, such as shape, rather than overall mass, and therefore indirectly influence peak area. This is understandable, given that rmHSP70 was only added to 0.1% of mAb content by mass. A summary of each run with the peaks for mAb1 and mAb2 monomer and aggregated proteins (with/without rmHSP70) are shown in Supplementary Fig. 3. The results provide additional support for the specific interaction of rmHSP70 with mAb aggregates.

**Table I:** Asymmetric Flow Field Flow Fractionation (AF^4^)^1^

#	#	Height^2^	Area	Elution time (min)
mAb1	Monomer	5	368.2	9.7
	rmHSP70-Monomer	4.9	347.8	9.7
	Aggregate	6	1152.8	21.2
	rmHSP70-aggregate	7.7	1413.6	21.5
mAb2	Monomer	4.4	288.3	10.3
	rmHSP70-Monomer	4.2	281.3	10.3
	Aggregate	4.4	217.8	6.7
	rmHSP70-aggregate	21	864.4	6.5

**Fig. 4:**
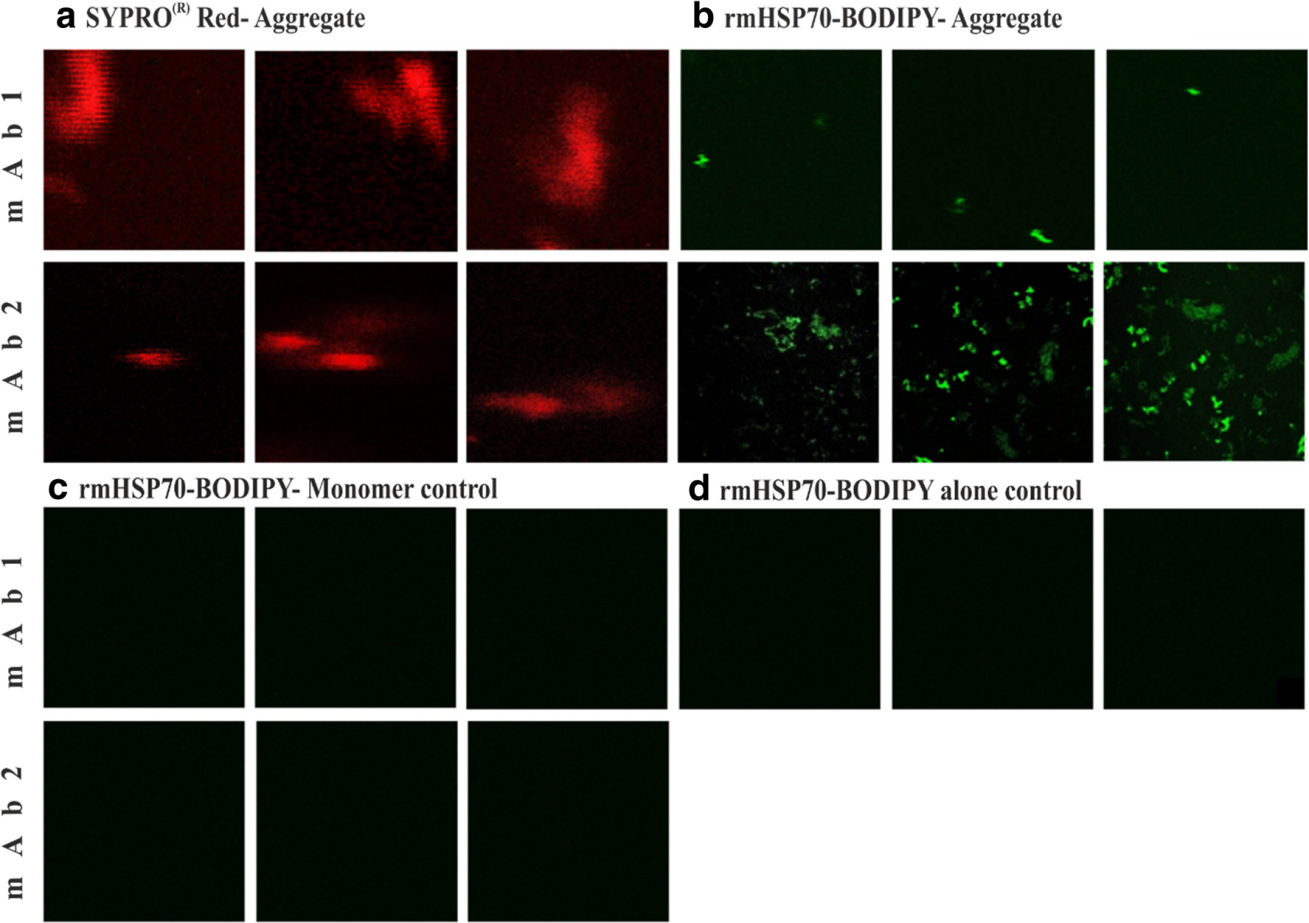
**Assessment of rmHSP70 binding to aggregated mAb using fluorescence imaging by RICS.** (**a**) SYPRO® Red dye stained aggregated samples of mAb1 and mAb2. (**b**) BODIPY FL tagged rmHSP70 (green) added to mAb1 and mAb2 post aggregation (0.1% by mass). (**c**) Monomer mAbs with rmHSP70-BODIPY FL. (**d**) rmHSP70-BODIPY FL alone. Data were obtained from a typical experiment using RICS mode and a 40x water immersion objective with slow scanning (pixel size 40 nm and scanning speed 6.4 microseconds per pixel.

The effect of rmHSP70 on the AF^4^ mAb aggregate profiles prompted us to investigate the interaction further by microscopy. rmHSP70 was labelled with the green fluorescence dye BODIPY and its binding to mAb1 and mAb2 aggregates studied. SYPRO® Red dye was added to mAb1 and mAb2 aggregates which were clearly visible by red fluorescence (Fig. 4a). Visualization of the BODIPY dye, for both mAb1 and mAb2, showed distinct concentration of the fluorophore in the presence of aggregates (Fig. 4b) but not in the presence of monomer (Fig. 4c) or absence of mAbs (rmHSP70-BODIPY alone control, Fig. 4d). It should be noted that mAb1 was used in a more diluted form than mAb2 in order to achieve optimal conditions for imaging, leading to the lower frequency of BODIPY-labelled rmHSP70-mAb1 aggregates observed in Fig. 4b. We infer that BODIPY-rmHSP70 bound to mAb aggregates, forming clumps which could be more readily visualized than when dispersed in bulk solution. These observations are therefore also consistent with specific adhesion of rmHSP70 to aggregates of mAb1 and mAb2.

### Effect of rmHSP70 on Immune Responses to mAb1 and mAb2 in BALB/c Model

*In vivo* approaches have been usefully adopted to compare immune responses elicited by aggregated proteins and their monomeric counterparts. A common approach is to measure antibody responses provoked in mice by immunizations with the monomeric or aggregated protein [33,34]. A BALB/c mouse model was therefore used in the current study to assess the immune responses generated following intraperitoneal (I.P.) immunization with monomer, rmHSP70-monomer, aggregated or rmHSP70-aggregated human mAbs [27].

**Fig. 5:**
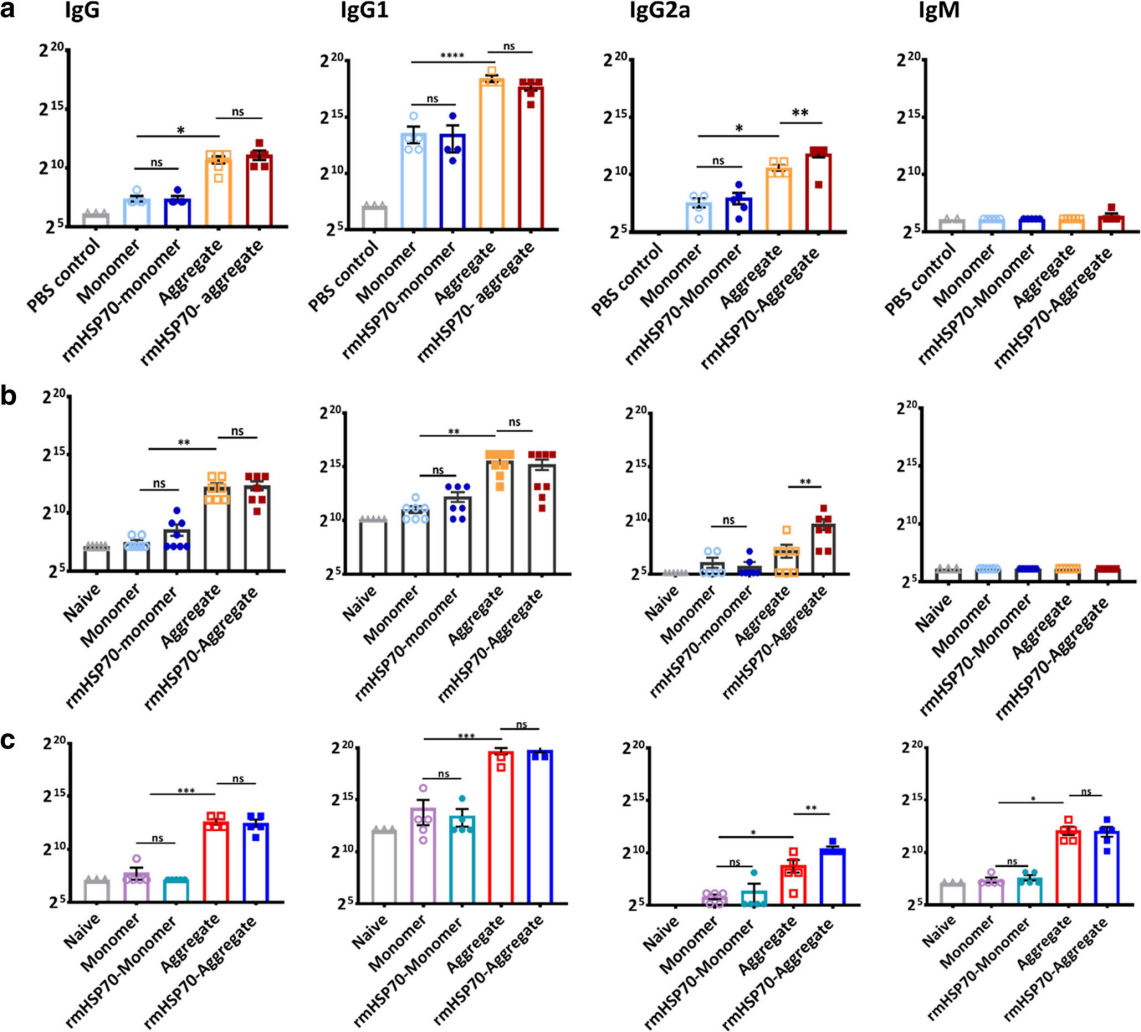
**Antibody responses to aggregated mAbs.** (**a**) mAb1 thermal stress-induced aggregates (**b**) mAb1 shaking stress-induced aggregates (**c**) mAb2 shaking stress-induced aggregates. BALB/c mice received monomer or aggregated protein injections (I.P.) with or without rmHSP70 addition. Mice received booster injections on day 7 and day 14, and were euthanized on day 21. Doubling dilutions (in 1% BSA/PBS) of serum samples from monomer, aggregate-immunized animals, PBS immunized or naïve negative control serum samples were analyzed against monomer-coated plates by ELISA for IgG, IgG1, IgG2a and IgM. Data are representative of two independent experiments for mAb1 (*n* = 8), and mAb2 (*n* = 5). ELISA titres for negative control mice (naïve or PBS) were invariably lower than the protein immunization groups (*p* < 001), symbols not shown. Differences in antibody serum titres between all sera groups against each substrate were analysed using one way ANOVA (*p < 0.05, **p < 0.01, ***p < 0.001).

mAb1 (250 μg) was administered as monomer or aggregate, both in the presence or absence of rmHSP70. Naïve mice or PBS only immunization was used as a sham control. Mice were boosted twice at 7 day intervals and were euthanized on day 21. Figure 5a shows the serum anti-mAb1 IgM, IgG and selected subclasses (IgG1, IgG2a) antibody responses following administration of thermal and shaking stress-induced aggregates. Levels of anti-mAb1 IgG antibody were elevated in the mice immunized with aggregate from thermal-stress and rmHSP70-aggregate mAb1 immunized mice, compared to those immunized with monomer mAb1, rmHSP70-monomer mAb1 or the PBS immunized control group (Fig. 5a). Similar patterns were recorded with anti-mAb1 IgG1 antibody responses but, due to the sensitivity of the anti-IgG1 detection antibody, these achieved considerably higher titres than those obtained for total IgG. Importantly, aggregation of mAb1enhanced the IgG2a antibody response compared to monomer and addition of rmHSP70 further enhanced IgG2a for the aggregate but not the monomeric species. These observations are indicative of a Th1-type skewing of the immune responses and consistent with those observed previously for an antibody fragment scFv [27]. The ability of mAb1 aggregates generated using shaking stress (1 μm) to induce immune responses in the BALB/c model was also examined. A very similar pattern was observed to the thermal stressed aggregates, with aggregate enhancing IgG and IgG1 antibody production and with rmHSP70 addition having a more profound effect on enhancing IgG2a antibody production than did aggregation alone (Fig. 5b). There was no detectable anti-mAb1-induced IgM antibody in response to mAb1 immunization (Fig. 5a, b).

In order to investigate whether these observations were particular to mAb1, mAb2 was investigated in a similar manner. Preliminary studies performed using mAb2 indicated that lower doses were needed to obtain measurable serum antibody responses in immunized mice (data not shown), and a dose of 150 μg mAb2 was used for the ensuing experiments. Mice immunized with shaking stress-induced mAb2 aggregates demonstrated increased IgG, IgG1, and IgG2a responses compared to immunization with monomer (Fig. 5c) and, as recorded for mAb1, rmHSP70 further enhanced IgG2a antibody production. A specific anti-mAb2 IgM antibody response was recorded for aggregate-treated mice, but not monomer. Similar patterns of antibody responses were observed in ELISAs for both monomer and aggregate-coated plates of mAb1 and mAb2.

**Fig. 6:**
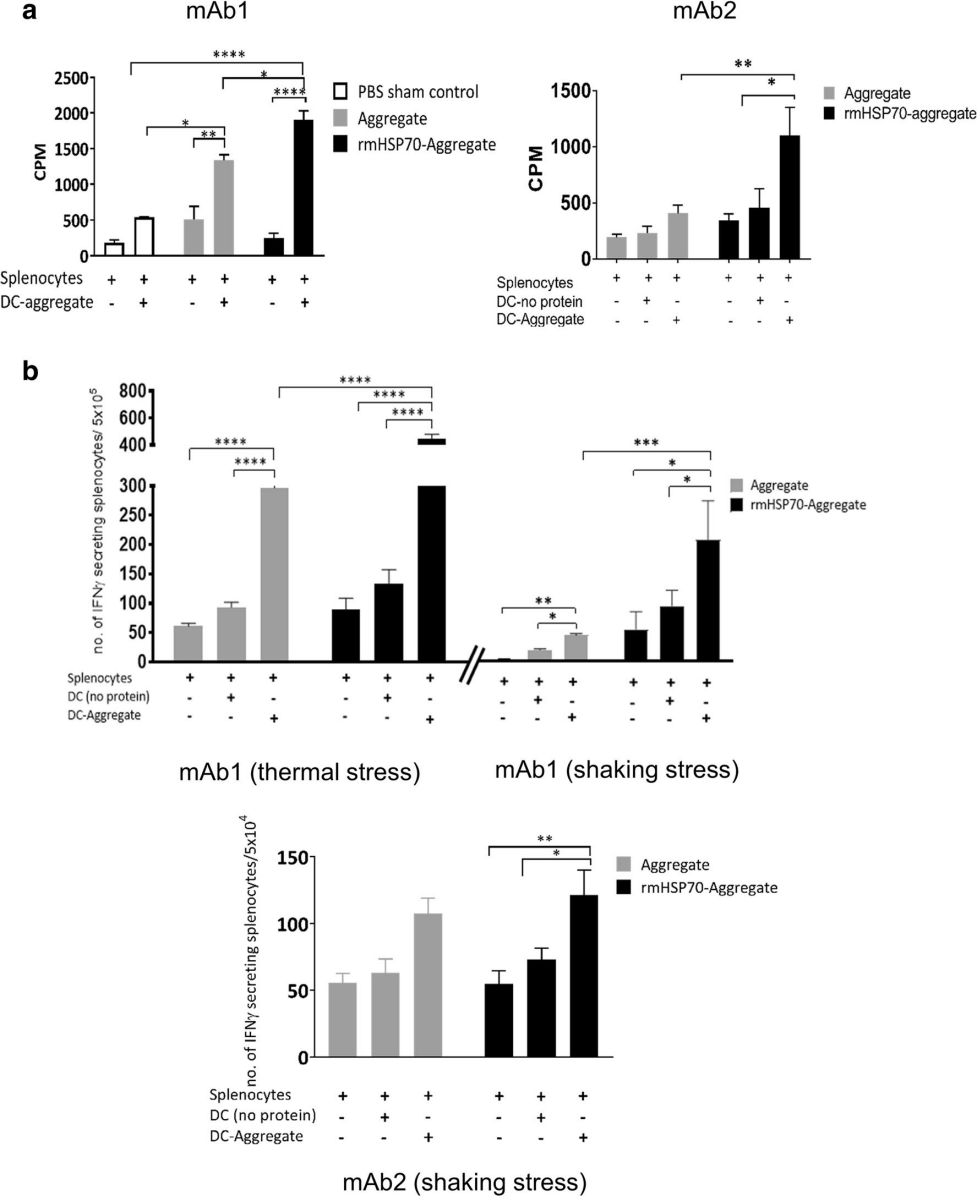
**Assessment of cellular responses in BALB/c mice immunized with aggregated mAbs in the presence or absence of rmHSP70.** (**a**) [^3^H] thymidine incorporation proliferation assay for aggregated mAb1 (thermal stress) and mAb2 (shaking stress). Splenocytes were isolated from immunized mice and stimulated ex vivo with protein. [^3^H] thymidine incorporation proliferation assays were performed by co-culturing *in vitro* protein-stimulated splenocytes from immunized mice with protein-pulsed DCs. A PBS only immunization was used as sham control. (**b**) IFNγ ELISpot assays. The IFNγ ELISpot assay was performed under the same culture conditions as (A) to assess IFNγ secretion in response to co-culture. The experiments were performed on two separate occasions, n = 8, (for thermal and shaking stress aggregates from mAb1) and n = 5 for mAb2. Comparisons of the means (± SEM) between groups are made. Statistical significance of the results obtained was calculated using one way ANOVA. *p < 0.05,**p < 0.01,***p < 0.001.

In order to further characterize adaptive immune responses induced in response to mAb immunization, splenocyte proliferation assays were conducted. Spleens were harvested on day 21 post-immunization and cultured ex vivo with addition of mAb1 or mAb2, with or without rmHSP70. The splenocytes were co-cultured with dendritic cells (DCs) from naïve BALB/c mice and mAb1 or mAb2; proliferation was measured with [^3^H]-thymidine incorporation. Statistically significant increases in proliferation were observed in the groups of mice that were immunized with rmHSP70-aggregate protein compared to mAb1 or mAb2 aggregate alone (Fig. 6a). IFNγ secretion ELISpot assays were performed by co-culturing *in vitro* protein-stimulated splenocytes with protein pulsed-DCs. For mAb1 (both thermal and shaking stress induced aggregates), a significantly higher frequency of IFNγ secreting splenocytes was observed in the group of mice immunized with rmHSP70-aggregate compared with aggregate alone. For mAb2, however, we did not observe any effect of rmHSP70 addition (Fig. 6b). Nevertheless, the cellular responses obtained were generally consistent with the antibody measurements, indicative of a Th1 enhancement in the groups immunized with aggregated mAbs, and provided further evidence for a stimulatory effect of rmHSP70 on immune responses to aggregated mAbs.

## Discussion

Protein aggregation can be described as the self-association of monomers in their native or partially unfolded forms [35,36], and is a common phenomenon observed in biopharmaceutical preparations. There is evidence that aggregates can stimulate an anti-drug immune response which may impair drug efficacy. However, the mechanisms through which immunogenicity is enhanced or conferred on proteins are only poorly understood [37]. Recently we reported that IgG2a antibody responses to an aggregated model scFv were stimulated in a mouse model by addition of a low (0.1%) level of a bacterial HSP, DnaK [27]. This observation led us to investigate whether this effect was also observed with human mAbs and a murine HSP. Aggregates that may be present in protein products can range from dimers to subvisible or visible particles. They can be formed during different stages of production, transport or delivery to the patient, in response to diverse stresses [35,38]^.^ Aggregates formed in biotherapeutic monoclonal antibodies under the influence of various stresses have been characterized by various techniques on the basis of their sizes, ranging from nm to micron dimensions [39–42]. The mAb aggregates employed in this study fall within this range and can therefore be regarded as typical, at least in terms of size, compared with those studied previously.

It is well established that HSP family members recognize exposed hydrophobic residues of misfolded or denatured proteins which are retained for ubiquitination and subsequent targeting to the proteasome for degradation [20,43]. Using co-sedimentation and western blotting, we have shown that rmHSP70 bound to aggregates of both mAbs. Similar phenomena were observed when aggregates were analyzed using AF^4^ where the presence of rmHSP70 had an effect on the migration profile of each mAb. Supporting these observations, microscopy demonstrated that fluorescently labelled rmHSP70 bound to the aggregated mAbs. Although the level of rmHSP70 used in these studies is high by industry standards (1:1000), our results provide evidence that HSP70 impurities can be selectively accumulated by adhesion to aggregates. This opens up the possibility that HSPs in general can selectively accumulate by adhesion to aggregated material, even though the overall levels of HSPs are low.

Our observations on IgG subclass specificity are consistent with those made previously by Ratanji *et al.* [27], which showed a stimulation of IgG2a levels characteristic of Th1-skewing of the immune response (Fig. 5). Interestingly, no increases in IgM antibody levels above PBS controls were detected for the immunizations of mAb1. This observation could be due to the immunogenic potential of mAb1, the serum IgM repertoire and the lifespan of IgM in the *in vivo* system [44]. Additional evidence for the stimulatory effects of rmHSP70 was obtained from cellular proliferation assays, using splenocytes cultured with antigen-primed DCs. Splenocyte proliferation was elevated in the group of mice immunized with rmHSP70-aggregated protein, compared with those immunized with aggregate alone, demonstrating an enhanced CD4-mediated T cell response. Stimulation of IFNγ secretion was particularly pronounced for mAb1 aggregates in splenocytes from the mice which received rmHSP70-aggregate, compared with aggregate alone. We think that the enhancement of mAb immunogenicity by rmHSP70 which we observe is unlikely to be attributable to its immunogenicity, for several reasons. First, the levels of rmHSP70 used were very low (0.1% of total protein inoculated). It is remarkable that such a low level of impurity can drive significant responses to IgG2a (Fig. 5) and splenocyte proliferation (Fig. 6a). Second, the *Cricetulus griseus* (Chinese Hamster) HSP70 is 98% identical to the mouse HSP70 sequence. Third, we do not observe an enhancement of IgG2a levels for monomer compared with rmHSP70-monomer, only for aggregate compared with rmHSP70-aggregate (Fig. 5).

Previous studies using mouse models have shown that HSP70 can be used as an adjuvant in cancer vaccine development strategies [45,46]. HSPs have therefore been harnessed as adjuvants of vaccines against cancers and infectious diseases [47]: examples include Phase I clinical trials for Glioblastoma (HSP70), colon carcinoma, Phase I-II studies for Non-small cell lung carcinoma (HSP70-activated NK cells) and a Phase I HIV vaccine study [25]. The adjuvant-like activity of HSP70 has been demonstrated *in vitro* by coupling to superparamagnetic iron oxide nanoparticles (SPIONs) to form HSP70-SPION conjugates. HSP70-SPIONs have shown effective delivery of immunogenic peptides from tumor lysates to DCs, stimulating a tumor-specific, CD8+ cytotoxic T cell response in experimental glioma models [48]. It is also known that HSP–peptide complexes can act as typical tumor-specific foreign antigens, chaperokines and adjuvants that facilitate uptake, processing, and presentation for tumor-specific antigens which are cross-presented by APCs to T lymphocytes [49]. The influence of HSPs on the induction of immunogenicity therefore appears to be a general phenomenon, rather than being confined to specific HSP-immunogen pairs.

HSP70 is released from cells in response to stress conditions [50]; more generally, intracellular proteins will be released by damaged, necrotic cells in a passive manner [51]. It is therefore not surprising that HSP family members have been recorded as HCP impurities in purified mAbs [15,52]. HSP70 is listed as a persistent HCP impurity in 29 mAb preparations following cross-interaction or Protein A affinity chromatography [53]. Accepted limits of HCP impurities are generally between 1 and 100 ppm [54]; ostensibly this would appear to be a low level but it does not take account of selective adsorption to aggregates, which would increase the apparent local concentration. Doses of therapeutic mAbs are well above 100 mg [55,56], so an impurity level of 0.1% of a particular HCP would result in administration of 100 μg, a level which could influence immunogenic responses to aggregates.

It has been reported that HSPs interact with and activate the immune system via Toll-like receptors on antigen-presenting cells [57,58]. It is also possible that aggregation itself enhances antigen uptake, and the presence of DnaK within the complex somehow enhances processing and presentation once inside the antigen-presenting cell. It is interesting to note that elevated levels of HSPs have been reported in the plasma of patients with certain illnesses such as dyslipidaemia [59], prostate cancer [60], and neurodegenerative diseases [61].

## Conclusion

These observations confirm and extend our previous work on DnaK/scFv [27], and provide additional evidence that the adjuvant-like effects of HSPs are general, rather than specific, to particular HSP/immunogen pairs. The precise mechanism(s) for the adjuvant effects of HSPs are currently a matter of debate, however. It is therefore difficult to predict which HSPs, or indeed other categories of HCPs, might act in this way and, if so, at what levels. Here we have used HSP70 as an exemplar HCP but our observations open up the possibility that a complex mixture of multiple HCPs may have synergistic effects on the immunogenicity of biotherapeutic mAbs. The implications for the immunogenicity of therapeutic mAbs in humans are harder to discern. We would argue that further work is needed to investigate the nature of this effect and determine the extent to which it contributes to the immunogenicity of aggregates of biopharmaceutical drugs.

## ACKNOWLEDGMENTS AND DISCLOSURES

We thank Lorna Beresford for her valuable support with animal work and Angela Thistlethwaite for her help with gel electrophoresis. We would also like to thank the Faculty Biomolecular Core Facility for DLS for instrument support and expertise. S. Rane was employed on a grant from MedImmune to carry out this research project. J. Derrick and the University of Manchester have received research grants from MedImmune. The other authors declare that they have no other relevant conflicts of interest.

### Funding Details

This work was funded by MedImmune.

## Electronic supplementary material

ESM 1 (DOCX 1111 kb)

## Abbreviations

ADA: Anti-drug antibody
AF^4^: Asymmetric flow field flow fractionation
APCs: Antigen presenting cells
BMDC: Bone marrow-derived dendritic cells
BSA: Bovine serum albumin
CHO: Chinese hamster ovary
DLS: Dynamic light scattering
HCP: Host cell protein
HRP: Horseradish peroxidase
IFNβ-1a: Interferon beta-1a
I.P.: Intraperitoneal
mAb: Monoclonal antibody
NMS: Normal mouse serum
PBS: Phosphate buffered saline
RICS: Raster image correlation spectroscopy
rmHSP70: Recombinant mouse heat shock protein 70
ROI: Region of interest
scFv: Single chain variable fragment
