# Supplementary material for: Impact of a Heat Shock Protein Impurity on the Immunogenicity of Biotherapeutic Monoclonal Antibodies
Source: Pharm Res. 2019 Feb 15;36(4):51. doi: 10.1007/s11095-019-2586-7 (PMC6394513; doi:10.1007/s11095-019-2586-7)
Supplement: Supplementary file 1 — (DOCX 1111 kb) [file 11095_2019_2586_MOESM1_ESM.docx]

Impact of a heat shock protein impurity on the immunogenicity of biotherapeutic monoclonal antibodies

**Shraddha S. Rane^a^, Rebecca J. Dearman^a^, Ian Kimber^a^, Shahid Uddin^b^, Stephen Bishop^c^, Maryam Shah^a^, Adrian Podmore^b^, Alain Pluen^a^, Jeremy P. Derrick^a^**

^a^School of Biological Sciences, Faculty of Biology Medicine and Health, Manchester Academic Health Science Centre , The University of Manchester, Michael Smith Building, Oxford Road, Manchester, M13 9PT, UK

^b^Medimmune Ltd, Granta Park, Cambridge, CB21 6GH, UK

^c^Medimmune, 1 Medimmune way, Gaithersburg, MD 20878, USA

Running title: Heat shock protein impurities and immunogenicity of biotherapeutic mAbs

To whom correspondence should be addressed: Prof. Jeremy P Derrick, Faculty of Biology, Medicine and Health, The University of Manchester, Michael Smith Building, Oxford Road, Manchester, M13 9PT, U.K., Telephone: +44 (0)161 306 4207, E-mail: [Jeremy.Derrick@manchester.ac.uk](mailto:Jeremy.Derrick@manchester.ac.uk)

**Keywords**

Monoclonal antibody, Aggregation, Host cell impurity, rmHSP70, Immunogenicity, Biotherapeutics, Innate Immunity, Anti-Drug Antibodies


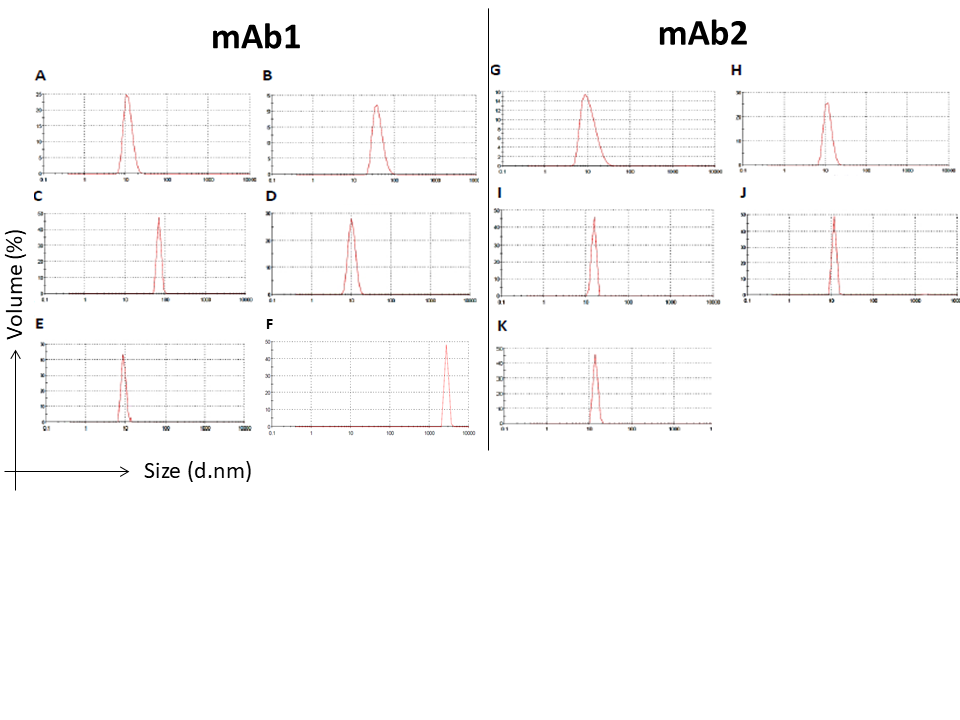


**Supplementary Figure 1: Analysis of mAb1 and mAb2 aggregates in response to different stresses using DLS.**

mAb1 samples: (A) mAb1 (10mg/mL, 22°C); (B) heat stressed (1mg/mL, 60°C, 25 min); (C) heat stressed (1mg/mL, 60°C, 25 min) and rested over night; (D) agitation stress (1mg/mL, 22°C, 24hr); (E) stir stressed (1mg/mL, 22°C, 16hr); (F) stir stressed (1mg/mL, 22°C, 96hr)

mAb2 samples: (G) heat stressed (1mg/mL, 45°C, 30min); (H) heat stressed (1mg/mL, 50°C, 30min); (I) heat stressed (1mg/mL, 60°C, 30min); (J) agitation stressed (1mg/mL, 22°C, 24hr); (K) heat stressed (10mg/mL, 60°C, 30min).

**A**

**
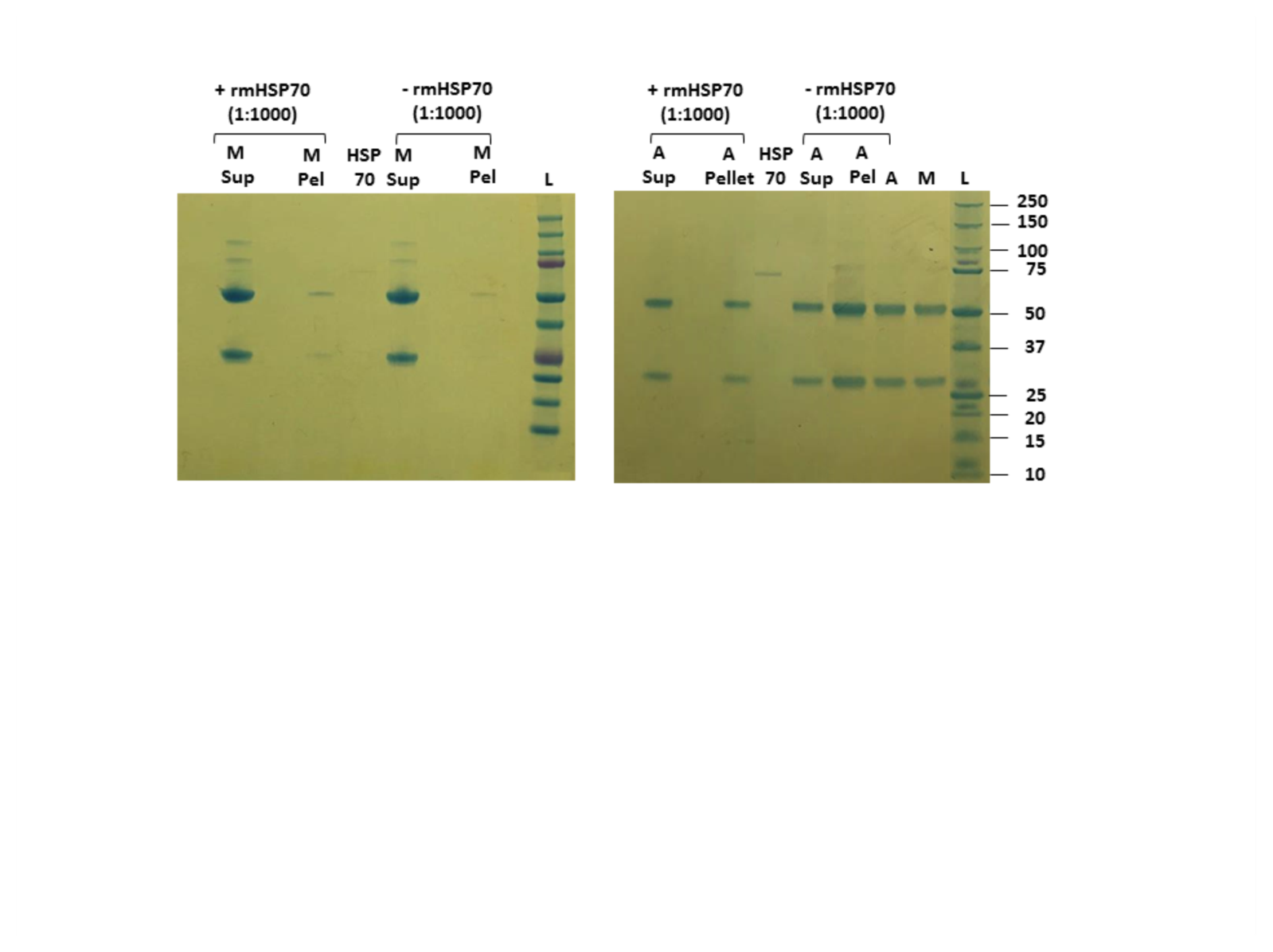
**

**B**

mAb1

**
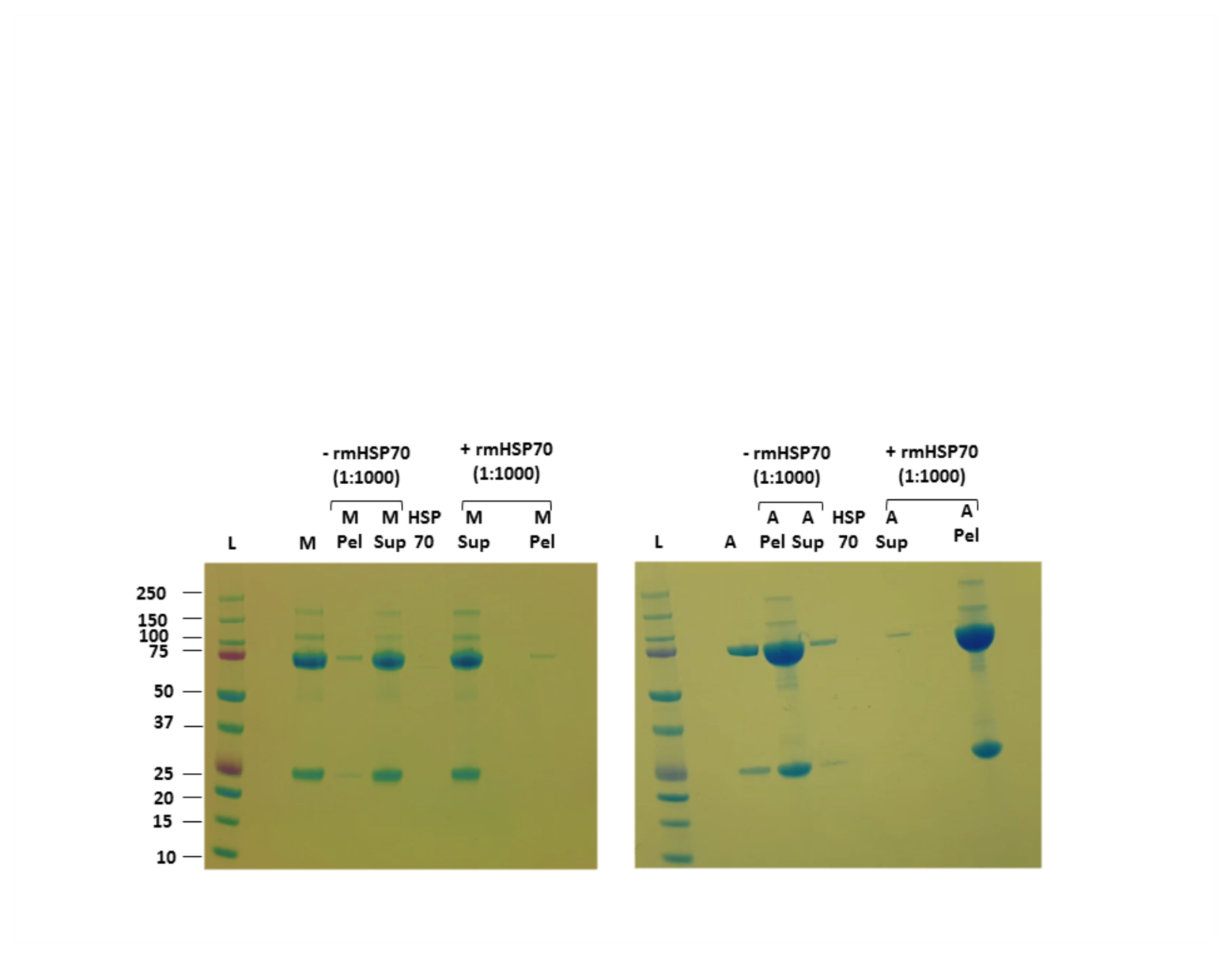
**

mAb2

**Supplementary Figure 2. SDS PAGE analysis of mAbs fractions, with/without rmHSP70.** A) mAb1 B) mAb2. mAb1 and mAb2 were aggregated using thermal and shaking stresses, respectively. rmHSP70 was added at 0.1% to monomer and aggregated mAbs immediately post aggregation. The samples were centrifuged to separate supernatant (Sup) and pellets (Pel) before loading on to the SDS PAGE. Gels were stained with Instant Blue. Samples were loaded on to the gels with and without rmHSP70 and labelled as M: monomer, A: aggregate, M Sup: monomer supernatant, M Pel: Monomer pellet, A Sup: aggregate supernatant, A Pel: aggregate pellet, HSP70: rmHSP70, L: molecular weight ladder marker. For mAb1 gels, lanes labelled 1, and 3 were left blank. For mAb2 gels, lanes labelled 2, 8, and 10 were left blank.

**B**

**A**

**
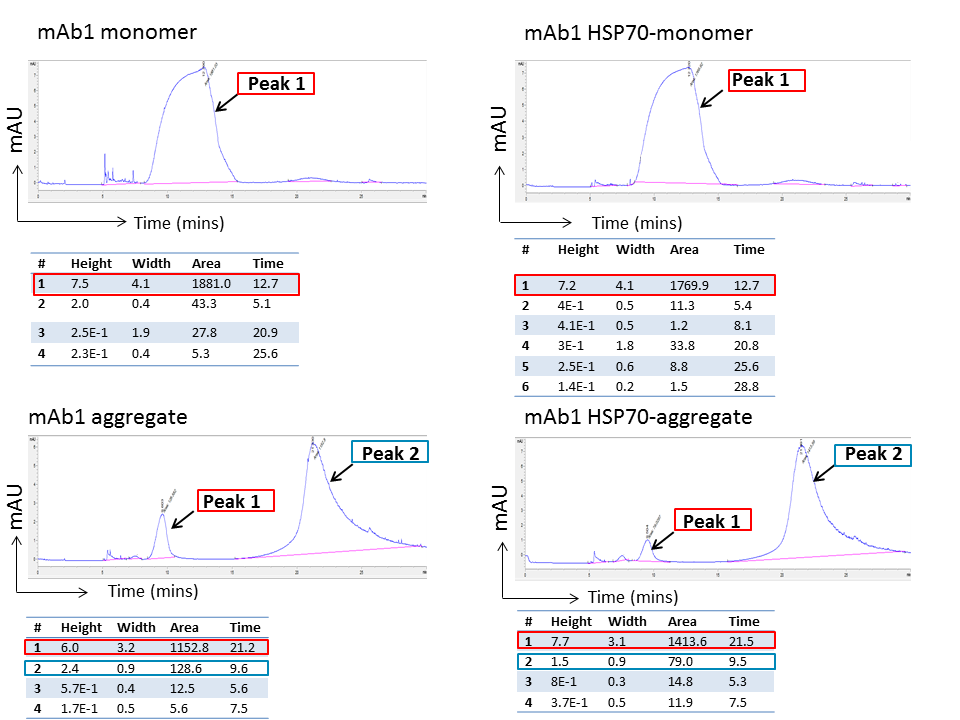
**

**F**

**E**

**D**

**C**

**
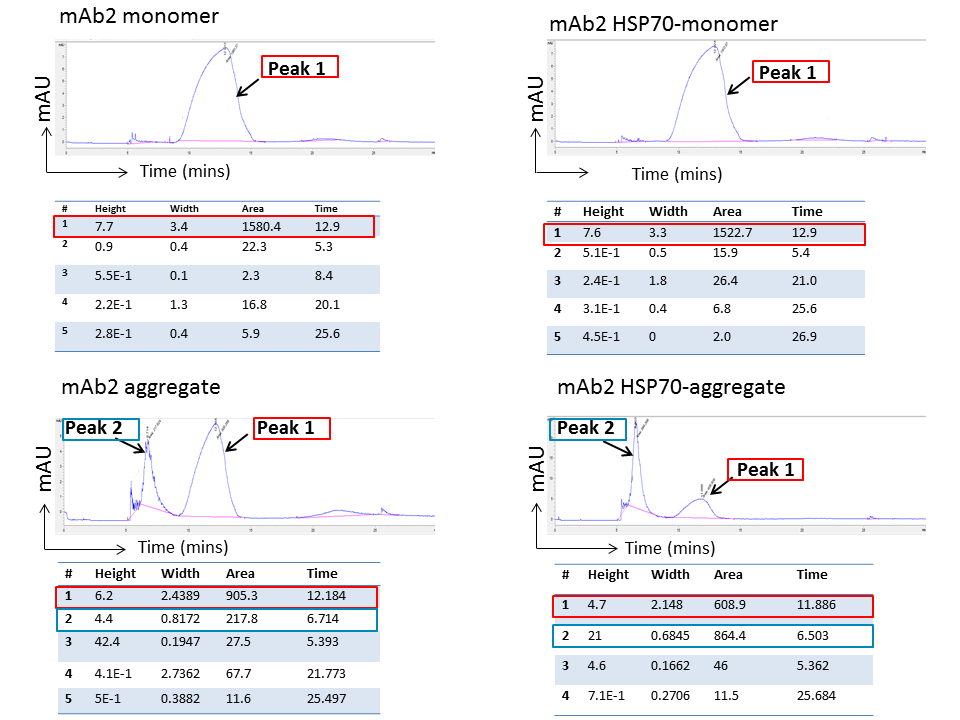
**

**H**

**G**

**Supplementary Figure 3.** Analysis of rmHSP70 binding to aggregated mAbs using AF^4^. mAb1 and mAb2 were aggregated using heat and shaking stresses, respectively. rmHSP70 was added to the aggregated mAbs immediately post aggregate formation (1:1000) and binding was assessed using AF^4^ method for height, width and total area of the peak over the period of sample run time (30min). The summary of each run is given below with the peaks for respective samples highlighted with boxes. Samples analyzed were: (A) mAb1 monomer, (B) mAb1 monomer with rmHSP70, (C) mAb1 aggregate, (D) mAb1 aggregate with rmHSP70, (E) mAb2 monomer, (F) mAb2 monomer with rmHSP70, (G) mAb2 aggregate, and (H) mAb2 aggregate with rmHSP70. Total area of the aggregate peaks for the mAbs with and without rmHSP70 was calculated and can be seen in the tables below with highlighted peak areas.
